# Variation in Carbon Storage and Its Distribution by Stand Age and Forest Type in Boreal and Temperate Forests in Northeastern China

**DOI:** 10.1371/journal.pone.0072201

**Published:** 2013-08-20

**Authors:** Yawei Wei, Maihe Li, Hua Chen, Bernard J. Lewis, Dapao Yu, Li Zhou, Wangming Zhou, Xiangmin Fang, Wei Zhao, Limin Dai

**Affiliations:** 1 State Key Laboratory of Forest and Soil Ecology, Institute of Applied Ecology, Chinese Academy of Sciences, Shenyang, China; 2 Tree Physiology Group, Swiss Federal Research Institute WSL, Birmensdorf, Switzerland; 3 University of Illinois at Springfield, Springfield, Illinois, United States of America; 4 University of Chinese Academy of Sciences, Beijing, China; DOE Pacific Northwest National Laboratory, United States of America

## Abstract

The northeastern forest region of China is an important component of total temperate and boreal forests in the northern hemisphere. But how carbon (C) pool size and distribution varies among tree, understory, forest floor and soil components, and across stand ages remains unclear. To address this knowledge gap, we selected three major temperate and two major boreal forest types in northeastern (NE) China. Within both forest zones, we focused on four stand age classes (young, mid-aged, mature and over-mature). Results showed that total C storage was greater in temperate than in boreal forests, and greater in older than in younger stands. Tree biomass C was the main C component, and its contribution to the total forest C storage increased with increasing stand age. It ranged from 27.7% in young to 62.8% in over-mature stands in boreal forests and from 26.5% in young to 72.8% in over-mature stands in temperate forests. Results from both forest zones thus confirm the large biomass C storage capacity of old-growth forests. Tree biomass C was influenced by forest zone, stand age, and forest type. Soil C contribution to total forest C storage ranged from 62.5% in young to 30.1% in over-mature stands in boreal and from 70.1% in young to 26.0% in over-mature in temperate forests. Thus soil C storage is a major C pool in forests of NE China. On the other hand, understory and forest floor C jointly contained less than 13% and <5%, in boreal and temperate forests respectively, and thus play a minor role in total forest C storage in NE China.

## Introduction

Temperate and boreal forests cover 1.9 billion hectares worldwide and account for approximately 46% of global forest carbon (C) storage [1]. Field and modeling studies suggest that these forests function as significant carbon sinks [2], [3], although the magnitude, location, and mechanisms of C sequestration remain uncertain [1], [4]. It is widely recognized that temperate and boreal forests are much more susceptible to global warming than tropical forests [5], [6], and that high northern hemisphere latitudes are experiencing a relatively rapid and significant change in climate [5]. The northeastern forest region of China (NE China) encompasses a forest area of more than 50×10^4^ km^2^, ranging from temperate forests in the south to boreal forests in the far north. These forests play an important role in the global carbon budget [7]. Thus a more thorough assessment of forest ecosystem C stocks and their dynamics in the country’s temperate and boreal forests is clearly worthwhile.

In northeastern China, numerous studies have been conducted to analyze spatial and temporal patterns of C storage on regional scales [8], [9], [10], to examine the effects of wildfire and human logging activities on changes in C storage [11], [12], [13], and to investigate C storage and its distribution across forest types via plot analyses [14], [15]. Such studies have advanced our knowledge of forest C storage and its variation at different scales. Stand age also has been shown to be a key factor in regulating C storage and its partitioning in different forest components (vegetation, debris and soil) [16], [17], [18]. To our knowledge, however, stand age has seldom been considered with respect to carbon dynamics and the pattern of carbon distribution in different forest components (tree, understory, forest floor and soil) in northeastern China.

The focus of this study therefore was to quantify the partitioning pattern of C storage in different forest components – tree, understory, forest floor and soil – across different aged forests (from young to over-mature) for the major natural temperate and boreal forests in NE China. The overall goal was to better understand C sequestration potential in boreal and temperate forests, and to provide information on carbon balances that might be used to improve forest management practices intended to increase carbon storage.

## Materials and Methods

### Ethics Statement

All necessary permits for the described field investigation were obtained at the start of the study from the provincial and locally state-owned forestry bureaus. The study forests refer neither to privately-owned field and biosphere nature reserves, nor to endangered or protected species.

### Study Area

**Figure 1:**
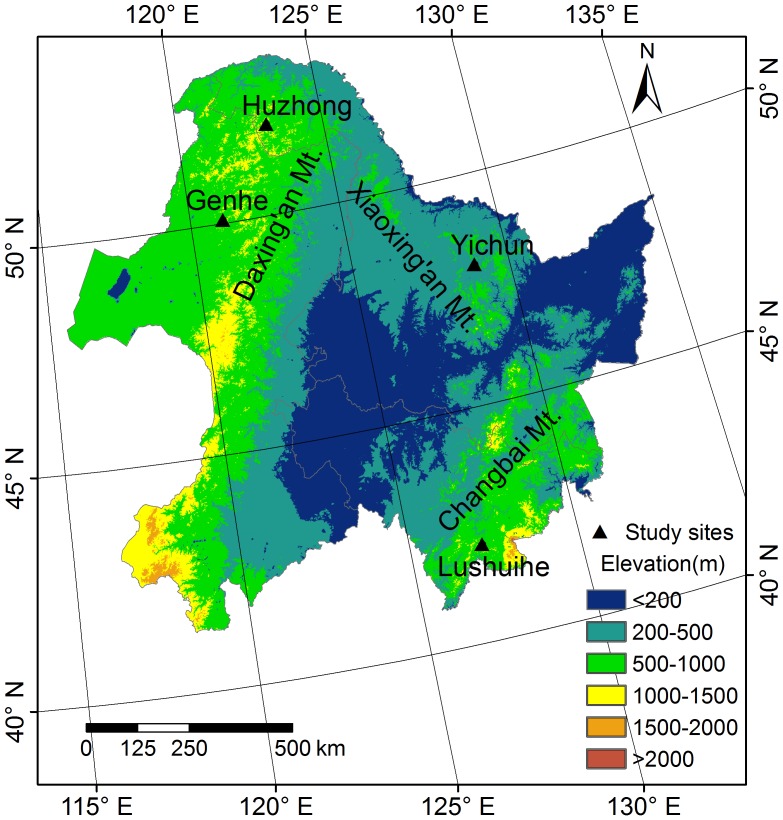
Geographic location of the study sites in the northeast forest region of China.

The study was conducted in state-owned forests in the northeastern forest region of China, which includes Heilongjiang and Jilin provinces and the eastern-most part of the Inner Mongolia Autonomous Region (41°42′∼53°34′N, 115°37′∼135°5′E, 109.04×10^4^ km^2^). Three major mountain ranges (Daxing’an, Xiaoxing’an and Zhangguangcai-Changbai) occur in the study region (Fig. 1). The climate is controlled by high latitude East Asian monsoons. Mean annual temperatures range from −2.5°C (north) to 4.8°C (south) and mean precipitation ranges from 250 mm (west) to1100 mm (east). From south to north, the forest region is divided into a temperate coniferous and broadleaved mixed forest zone (Changbai and Xiaoxing’an mountains), and a boreal coniferous zone (Daxing’an mountain range). Dark-brown soils are predominant in the temperate zone and brown coniferous forest soils in the boreal zone.

### Field Design

Four representative sites in NE China forests were selected for study – the Lushuihe site in the Changbai mountain area; the Yichun site in the Xiaoxing’an mountains; and the Genhe and Huzhong sites in the Daxing’an mountains (Fig. 1). The Lushuihe and Yichun sites include three major temperate natural forest types – coniferous mixed forest (CMF), coniferous and broadleaved mixed forest (CBF), and broadleaved mixed forest (BMF); While the Genhe and Huzhong sites include two major boreal natural forest types – larch forest (LF) and birch forest (BF) (Table 1). In each type, four stand age classes were delineated according to the ages of dominant trees. For CMF, CBF, and LF, age classes were defined as young (<40 years), mid-aged (41–80 years), mature (81–140 years), and over-mature (>141 years). For BMF and BF forests, stand ages were defined as young (<30 years), mid-aged (31–50 years), mature (51–80 years), and over-mature (>81 years). During the field investigation, stand ages were based on the predominant tree species and other information (i.e. forest maps, forest management or logging history, and so on) provided by local forestry bureaus.

**Table 1:** Characteristics of the study sites and stands in northeastern China.

	Geographic factors			Climatic factors		Community characteristics			
Sites	Latitude(N)	Longitude (E)	Elevation (m,asl)	MAT (°C)^a^	MAP (mm)	Forest types^b^	Dominant tree species	Stand density (trees·ha^−1^)	No. of plots
**Boreal zone**									
Huzhong	51°4′∼52°2′	122°2′∼124°0′	446∼990	−0.8∼1.1	359∼636	LF, BF	*Larix gmelinii*, *Betula platyphylla*	1000–2567	38
Genhe	50°3′∼50°6′	120°6′∼121°3′	446∼1011	−2.5∼−0.7	208∼381	LF, BF	*Larix gmelinii*, *Betula platyphylla*	1353–3460	41
**Temperate zone**									
Yichun	47°1′∼48°2′	128°1′∼129°2′	259∼599	1.2∼2.8	421∼823	CMF,CBF, BMF	*Pinus koraiensis, Picea jezoensis, Abies nephrolepis, Populus davidiana, Tilia amurensis, Quercus mongolica*	613–2644	57
Lushuihe	42°3′∼42°4′	127°5′∼128°0′	582∼1039	2.6∼4.8	509∼810	CMF,CBF, BMF	*Pinus koraiensis, Picea jezoensis, Abies nephrolepis, Populus davidiana, Tilia amurensis, Quercus mongolica*	650–1663	43

a MAT = mean annual temperature; MAP = mean annual precipitation.

b Forest types: LF: larch forest; BF: birch forest; CMF: coniferous mixed forest; BMF: broadleaved mixed forest; CBF: coniferous and broadleaved mixed forest.

Each of the 179 study plots was 20×20 m (Table 1). Tree biomass, understory biomass, forest floor biomass and soil C were measured within each plot. A small subset of plots within forest types of the same or similar age class, species composition, and geographical conditions served as replicates within each age class and forest type replicated three times.

### Field Sampling and Forest Carbon Storage Estimation

#### Trees

Within each 400 m^2^ plot, all trees (standing and fallen) with a diameter at breast height (DBH, 1.37 m above ground) of ≥5 cm were identified in terms of species, height, DBH, and living or dead status. Individual tree biomass (above and below-ground) was estimated using species-specific allometric equations, developed by Chen & Zhu [19] for the Changbai area, Wang [20] for the Xiaoxing’an area, and Han & Zhou [21] for the Daxing’an area. For a few tree species where no species-specific allometric equations were available, we used equations for similar species. Given that such trees were seldom encountered, the influence of surrogate equations was considered insignificant.

#### Understory

Understory vegetation included shrubs, herbs, and small trees with a DBH <5 cm. This component was measured in three randomly established 2×2 m subplots within each plot. All vegetation in subplots was harvested and weighed with an electronic balance (accuracy: ±1 g). The fresh weight was recorded for small trees (foliage, branches and stems), shrubs (leaves and branches) and herbs. Understory biomass was estimated via fresh weight multiplied by previously established dry-wet ratios for different understory vegetation in NE China [22].

#### Forest floor

For this study we defined forest floor biomass as woody debris, surface litter, organic matter above the mineral soil, and undifferentiated organic matter. Only fine woody debris, snags and fallen wood were measured, since coarse woody debris (CWD) with a mid-length diameter >2.5 cm had been almost completely removed by local farmers. All of these components were collected in three randomly selected 1×1 m quadrats within each plot, and their fresh weights obtained with an electronic balance (accuracy: ±1 g). Subsamples of fresh weights were taken to the laboratory and oven-dried at 65°C to constant weight (0.1 g) to obtain fresh mass/dry mass ratios for calculating the dry mass of all samples. The oven-dried samples were also utilized to measure organic carbon content using the K_2_Cr_2_O_7_-Oxidation method [23].

#### Soil C storage

Soil samples were obtained from two randomly selected vertical profiles within each 20×20 m plot. Soils were sampled to depths that either reached the parent material or did not exceed 1 m. Each soil profile was divided into the following vertical layers of 0–10, 10–20, 20–30, 30–50 and 50–100 cm. In this study, the average soil profile depth for the temperate zone forests was approximately 100 cm and that of the boreal zone forests was 40 cm (Table 2). For each plot, soil samples were extracted and mixed in order to obtain a 0.5-kg sample for each layer. Soil cores (100 cm^3^, 5.0 cm in diameter and 5.0 cm in depth) were collected for bulk density (BD) estimation. Rocks and gravel (>2 mm in diameter) were sieved and their content (%) estimated for each soil layer. When BD could not be measured directly due to a large amount of stones, it was estimated from adjacent additional profiles within the plot.

**Table 2:** Soil bulk density, soil C content and soil C density in boreal and temperate forest soils (mean ±1SD) in northeastern China.

	Boreal forests				Temperate forests			
		Soil property				Soil property		
Soil depth (cm)	No. ofsamples	Bulk density(g·cm^−1^)	C content(g·kg^−1^)	C density(MgC·ha^−1^)	No. ofsamples	Bulk density(g·cm^−1^)	C content(g·kg^−1^)	C density(MgC·ha^−1^)
0–10	147	0.85±0.26	64.5±3.50	50.6±16.33	197	0.66±0.17	73.1±2.43	46.3±13.51
10–20	118	1.16±0.28	33.9±1.89	31.9±18.45	197	1.06±0.19	32.7±1.58	33.4±13.62
20–30	56	1.32±0.30	21.5±1.39	23.4±13.66	188	1.27±0.21	19.6±1.01	23.9±11.51
30–50	10	1.41±0.13	18.1±1.01	33.1±27.19	179	1.41±0.18	12.5±0.75	29.6±15.72
50–100	5	1.35±0.10	14.9±0.42	71.6±20.35	107	1.51±0.17	7.9±0.51	45.9±30.92

Soil organic C content was determined using the K_2_Cr_2_O_7_-Oxidation method [23]. Soil organic C was estimated from the following equation [24]:

where SOC is the total soil organic C storage (Mg·C·ha^−1^) of a given profile; SOC*_i_* is the SOC content (g·kg^−1^) in soil layer *i*, BD*_i_* is the bulk density (g·cm^−3^) in soil layer *i*, H*_i_* is the thickness (cm) in the soil layer *i*, and R*_i_* is the volumetric fraction (%) of stones >2 mm in the soil layer *i* (Table 2).

### Data Analysis

Tree biomass C storage was calculated as the product of biomass multiplied by carbon conversion coefficients, which for NE China range from 0.49 for broadleaved mixed forest to 0.52 for larch forest [25]. Understory biomass C storage was obtained by utilizing the standard biomass-C storage transformation coefficient of 0.5 [26], and forest floor biomass C was estimated through its organic C content. Total C storage was the sum of tree biomass, understory biomass, forest floor biomass, and soil C storage.

Statistical analyses were conducted using SPSS 16.0 software. Two-way ANOVA was used to test for effects of forest zone (boreal vs. temperate) and stand age on C storage by total C, tree C, understory C, forest floor C, and soil C. Within each forest zone, effects of forest type and stand age on C storage and its proportion in different components were tested using two-way ANOVA, followed by one-way ANOVA (LF vs. BF) or Tukey’s HSD test, to compare the means across the four stand age classes. Significance levels were set at P<0.05 for all analyses.

## Results

### Carbon Storage across Forest Zones

Carbon storage was strongly affected by forest zone and stand age (Table 3). Total C, tree C, forest floor C and soil C storage all differed with forest zone (all P-values were <0.001; Table 3), with the exception of understory C (F = 0.30, P = 0.59; Table 3). Stand age significantly affected total C, tree C, and forest floor C but not understory C and soil C storage (Table 3). There were significant interactions between forest zone and stand age associated with tree C and forest floor C storage (both interactions with P<0.001, Table 3).

**Table 3:** Effects of forest zone and stand age on forest C storage in northeastern China.

		Total		Tree		Understory		Forest floor		Soil	
Factors	df1/df2	F-value	P	F-value	P	F-value	P	F-value	P	F-value	P
Forest zone (Z)	1/177	44.06	0.00	48.51	0.00	0.30	0.59	178.25	0.00	16.80	0.00
Age class (A)	3/175	43.47	0.00	93.54	0.00	1.39	0.25	15.39	0.00	0.51	0.67
Z×A	3/171	1.49	0.22	9.09	0.00	0.73	0.54	7.38	0.00	0.45	0.72

Note: df1 and df2 are the numerator and denominator degrees of freedom, respectively. Statistical significances were tested using two-way ANOVA based on F-values; a P value of <0.05 indicates significance of differences at the 0.05 level. The two forest zones are boreal zone and temperate zone; the four stand age classes are young, mid-aged, mature, and over-mature.

### Carbon Storage in Boreal Forests

**Figure 2:**
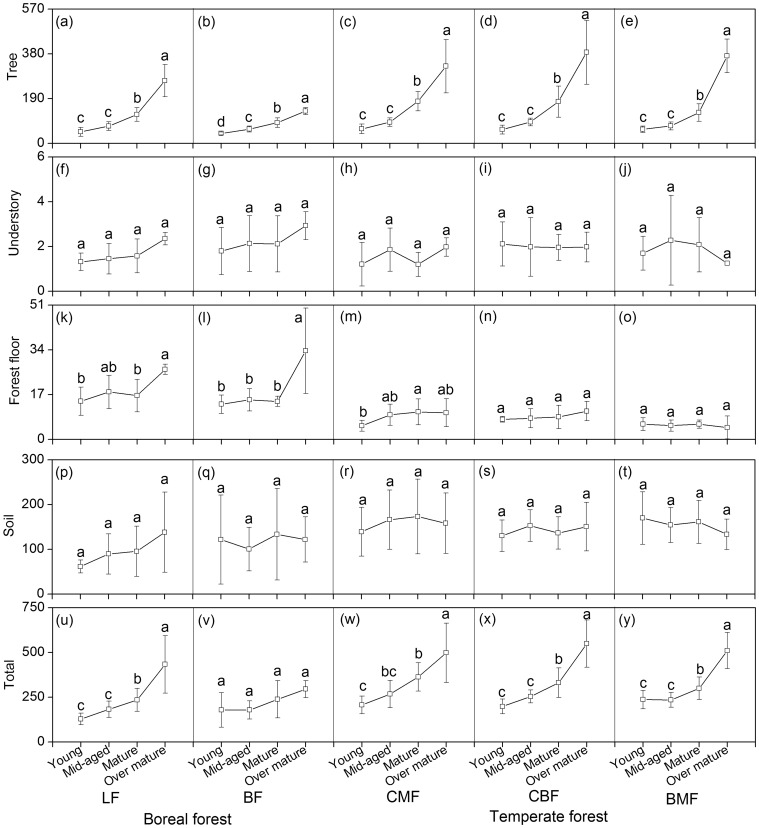
C storage (mean values ±1SE, Mg·C·ha^−1^) in young, mid-aged, mature and over-mature stands in the boreal and temperate forest zones in northeastern China. Notes: (1) Forest types in boreal forest zone: larch forest (LF); birch forest (BF); Forest types in temperate forest zone: coniferous mixed forest (CMF); coniferous and broadleaved mixed forest (CBF); broadleaved mixed forest (BMF). (2) Different letters within each cell indicate significant differences among the four age classes.

**Figure 3:**
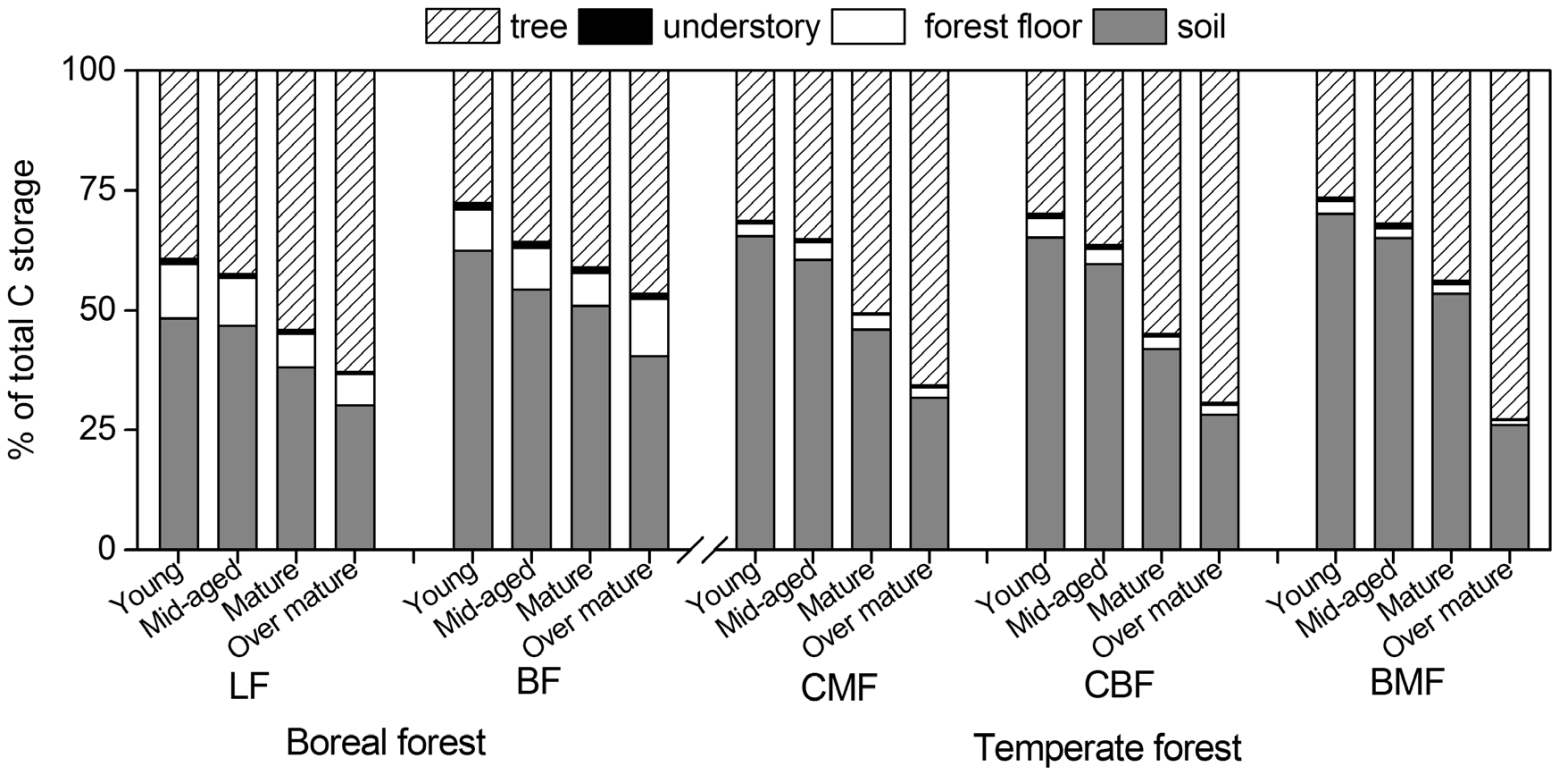
Distribution pattern of C storage among forest components in young, mid-aged, mature, and over-mature stands in boreal and temperate forests of northeastern China. Forest types in boreal forest zone: larch forest (LF); birch forest (BF). Forest types in temperate forest zone: coniferous mixed forest (CMF); broadleaved mixed forest (BMF); coniferous and broadleaved mixed forest (CBF).

In the boreal forests, forest type was significantly correlated only with tree C, whereas stand age was significantly correlated with tree C, forest floor C, and total C storage (Table 4). The latter increased with increasing stand age, with highest storage levels (295.8–434.0 Mg·C·ha^−1^) occurring in over-mature forests (Fig. 2u, v). Tree biomass C was significantly greater in LF (49.3–266.7 Mg·C·ha^−1^) than in BF (42.4–137.0 Mg·C·ha^−1^) (Fig. 2a, b; Table 4). The contribution of tree biomass C to total C storage varied significantly by forest types and stand age (Table 4). Percentage C in trees ranged from 27.7% (young) to 46.6% (over-mature) for BF, and from 39.3% (young) to 62.8% (over-mature) for LF (Fig. 3).

**Table 4:** Effects of forest type and stand age on C storage by forest components (tree, understory, forest floor and soil) and their percent of total C storage in boreal and temperate forests in northeastern China.

		Boreal forest^a^					Temperate forest				
			C storage		C percent			C storage		C percent	
Components	Factors	df1/df2^b^	F-value	P^c^	F-value	P	df1/df2	F-value	P	F-value	P
Tree	Forest type (F)	1/78	48.80	**0.00**	10.84	**0.00**	2/97	0.88	0.42	1.21	0.30
	Age class (A)^d^	3/75	73.90	**0.00**	7.06	**0.00**	3/96	86.03	**0.00**	65.80	**0.00**
	F×A	3/71	10.29	**0.00**	0.44	0.72	6/88	1.01	0.43	0.84	0.54
Understory	Forest type (F)	1/78	3.69	0.06	3.09	0.08	2/97	1.06	0.35	0.59	0.56
	Age class (A)	3/75	1.41	0.25	0.68	0.57	3/96	0.55	0.65	3.12	**0.03**
	F×A	3/71	0.04	0.99	0.16	0.92	6/88	0.58	0.75	0.46	0.83
Forest floor	Forest type (F)	1/78	0.01	0.92	0.21	0.65	2/97	7.26	**0.00**	5.46	**0.00**
	Age class (A)	3/75	10.48	**0.00**	5.28	**0.00**	3/96	1.62	0.19	4.10	**0.00**
	F×A	3/71	1.09	0.36	2.59	0.06	6/88	1.20	0.32	0.99	0.44
Soil	Forest type (F)	1/78	1.26	0.27	7.43	**0.00**	2/97	0.80	0.45	1.70	0.19
	Age class (A)	3/75	0.77	0.51	3.82	**0.01**	3/96	0.28	0.84	53.29	**0.00**
	F×A	3/71	0.65	0.58	0.28	0.84	6/88	0.49	0.81	0.63	0.71
Total	Forest type (F)	1/78	1.01	0.32			2/97	0.20	0.82		
	Age class (A)	3/75	12.42	**0.00**			3/96	41.98	**0.00**		
	F×A	3/71	1.95	0.13			6/88	0.87	0.52		

a Boreal forests are composed of larch and birch; temperate forests include coniferous mixed forest, broadleaved mixed forest, coniferous and broadleaved mixed forest.

b Refers to numerator (df1) and denominator (df2) degrees of freedom, respectively.

c Statistical significances was tested using two-way ANOVA; a P value of <0.05 indicates significance of difference at the 0.05 level.

d Stand age classes are young, mid-aged, mature, and over-mature.

Understory biomass C ranged from 1.3 to 2.9 Mg·C·ha^−1^ across age classes and forest types (Fig. 2f, g, Table 4). It accounted for only a small proportion of total C storage (0.6%–1.3%) (Fig. 3).

Forest floor biomass C in over-mature forests (up to 26.6–33.7 Mg·C·ha^−1^; Fig. 2k, l) was significantly greater than that in any other age classes (P<0.01, Table 4). The proportion of forest floor C to total C storage was significantly correlated with stand age and ranged from 6.5%–12.0% (Table 4; Fig. 3).

Both soil C content and density (per 10 cm soil layer) decreased with soil profile depth, whereas soil total C storage increased with depth (Table 2). Soil C storage did not vary significantly with either forest type or stand age (Fig. 2p, q; Table 4). However, its contribution to total C storage significantly differed among forest types and significantly decreased from 62.5%–48.4% in young forests to 40.4%–30.1% in over-mature stands (Fig. 3; Table 4).

### Carbon Storage in Temperate Forests

In temperate forests, forest type was significantly related only to forest floor C whereas stand age significantly affected both total C and tree C storage (both P<0.001, Table 4). Total C storage was greatest in the over-mature forests (498.5–549.8 Mg·C·ha^−1^) (Fig. 2w–y). Tree C storage increased with increasing stand age (P<0.05, Fig. 2c–e), resulting in a significant positive correlation between percent of tree C contributions to total C storage and stand age (Table 4); percentages ranged from 26.5%–31.3% in young stand to 65.7%–72.8% in over-mature stands (Fig. 3).

Understory C, which ranged from 1.2 to 2.3 Mg·C·ha^−1^, did not vary significantly with either stand age or forest type (Fig. 2h–j, Table 4). In contrast, its contribution to total C storage (0.3%–1.0%) decreased significantly with increasing stand age (Fig. 3; Table 4).

Forest floor C was significantly affected by forest type (Table 4), with lowest values occurring in BMF (Fig. 2o). The contribution of forest floor C to total C storage (ranging from 1.0% to 4.1%) varied significantly with both forest type and stand age (Fig. 3, Table 4).

As in the boreal forest, both soil C content and density decreased with soil profile depth (Table 2), but soil C did not vary significantly with forest type or stand age in the temperate forests (Table 4; Fig. 2r–t). At the same time, soil C contribution to total C storage decreased significantly with stand age (Table 4), and ranged from 65.2%–70.1% in young forests to 26.0%–31.7% in over-mature forests (Fig. 3).

### Carbon Storage in the 0–20 cm Soil Layer

**Figure 4:**
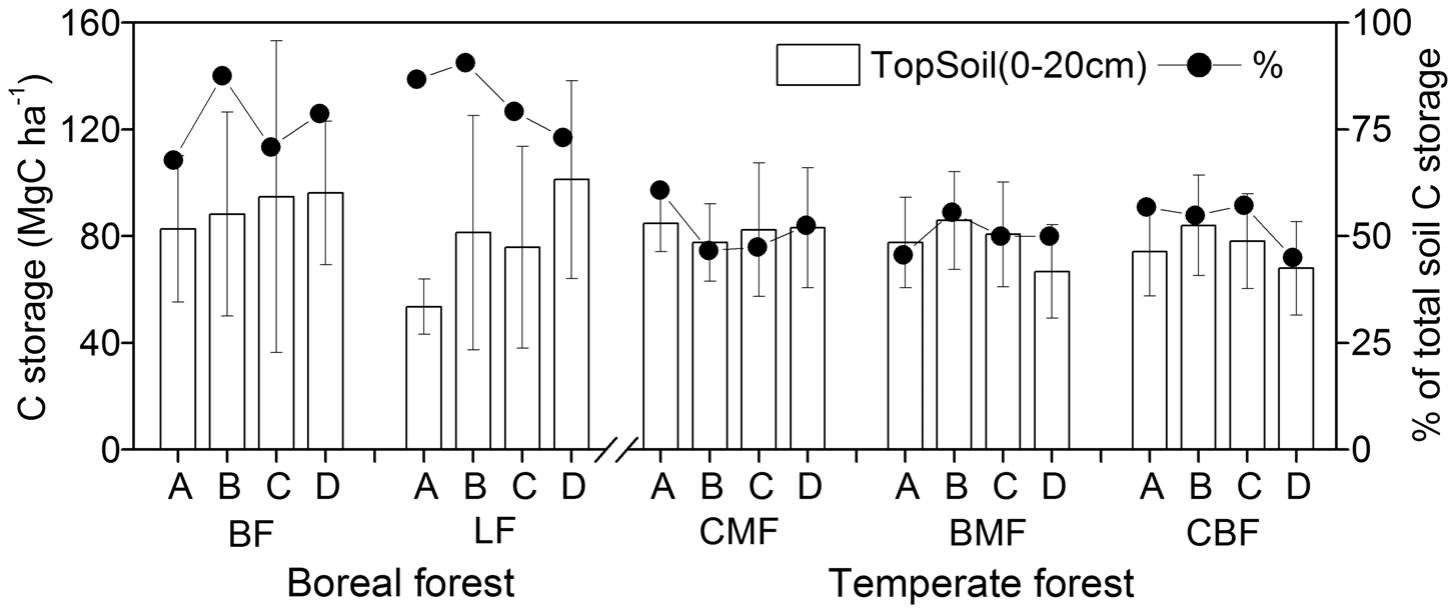
C storage in 0–20 cm soil layers (mean values ±1SE, Mg·C·ha^−1^) and its contribution to total soil C storage across four stand age classes in boreal and temperate forests in northeastern China. Forest types in boreal forest zone: larch forest (LF) and birch forest (BF). Forest types in temperate forest zone: coniferous mixed forest (CMF), broadleaved mixed forest (BMF), coniferous and broadleaved mixed forest (CBF). Age classes: young (A), mid-aged (B), mature (C), over-mature (D).

C storage in the top 20 cm of the soil did not vary significantly among forest types or stand ages. It ranged from 53.6 to 101.1 Mg·C·ha^−1^ in boreal forests and from 66.8 to 85.8 Mg·C·ha^−1^ in temperate forests (Fig. 4). Its contribution to total soil C storage also did not vary significantly between forest types or among stand ages (Fig. 4), and accounted for 67.8%–90.6% in boreal forests and 45.0%–60.8% in temperate forests.

## Discussion

### Carbon Storage in Temperate vs. Boreal Forests

With the exception of understory C, carbon storage of other forest components (tree, forest floor, and soil), as well as total C varied with forest zone (temperate vs. boreal) which is associated with climate conditions (Table 3). Thus, for example, tree C in temperate forests exceeded that in boreal forests (Fig. 2a–e), possibly due to greater forest ecosystem net primary productivity (NPP) associated with higher temperature and a longer growing season in the former [27], [28], given the general absence of water deficit in NE China [9], [29]. Previous studies conducted in other regions also found that biomass C storage in temperate forests exceeded that of tropical and boreal forests, leading to the conclusion that cool temperatures in combination with moderate precipitation favors biomass carbon accumulation [30], [31]. Moreover, the relatively high plant species diversity or tree species composition in the temperate mixed forests compared to the boreal forests may also lead to greater biomass productivity and accumulation in the former in our study region [32].

Forest floor biomass is determined by the net balance between litter fall input and decomposition output. Climate can influence forest floor biomass by controlling the rates of these two processes [33], [34]. Although variables such as forest type, stand age and disturbance regime are important in controlling forest floor biomass [35], the greater level of forest floor C in boreal as opposed to temperate forests (Figs. 2k–o; Table 3) suggests that low temperature plays a more important role than these other factors in determining forest floor biomass in NE China [34].

Soil C storage decreased from temperate forests to the more northern boreal forests (Fig. 4d), which is inconsistent with the findings of previous studies [36], [37]. However, our results may have been influenced by specific edaphic factors that differed across the two forest zones. Others have found that soil profile total C storage increases with increasing soil thickness [24], [38]. The relatively shallow soils of the boreal forest (approximately 40 cm) compared to the temperate forests (approximately 100 cm) (Table 2) is the likely cause of the lower soil C storage level in boreal forests. Correspondingly, the ratio of total forest C to total soil C in the upper soil layers (0–20 cm) was higher in boreal forests (67.8%–90.6%) than in temperate forests (45.0%–60.8%) (Fig. 4). However, these fractions are still much lower than those found in other boreal forests [16], [39], while our results may have been influenced by the relatively large proportion of stones and shallower soils in the boreal forests we observed.

Total C storage was much higher in temperate forests (198.9–549.8 Mg·C·ha^−1^) than in boreal forests (128.6–434.0 Mg·C·ha^−1^), which agrees closely with the regional-scale study results of Pregitzer et al. [37] (239 vs. 143 Mg·C·ha^−1^). The greater C storage in temperate forests was related to the high tree biomass and soil carbon storage as mentioned above. Similarly, large C storage occurred in the temperate forests of the Pacific Northwest Region of North America [30], [31]. But on a global scale, Pan et al. [1] showed that the average total C storage in boreal forests (239 Mg·C·ha^−1^) exceeded that of temperate forest (155 Mg·C·ha^−1^). Such observed differences may be the result of study region size and heterogeneity and related issues involving scaling that can, by themselves, produce uncertainty and high spatial variation in forest ecosystem C storage. Such differences also may be related to climatic and edaphic factors, human disturbance, and stand age structure [4], [31], [37].

### Effects of Forest Type on Carbon Storage

Neither total C storage nor that of forest components (tree, understory, forest floor and soil) varied significantly with forest type, with the exception of tree C in boreal forests and forest floor C in temperate forests (Table 4). Given that tree C significantly differed by forest type in boreal but not temperate forests may be associated with differences in tree species composition or species diversity [32]. In our study, boreal forests formed nearly pure stands with relatively low species diversity, unlike the temperate mixed forests we observed with their higher species diversity (Table 2). Although forest type and tree species composition have been found to influence both forest floor C and soil C storage through litter production, litter quality, and the surrounding decomposition environment [40], [41], human disturbance also can play a role in regulating C storage in the forest floor and soil [42]. Bunker et al. [32] and Saha et al. [43] nevertheless reported that forest type and tree species composition are important in regulating potential C storage even when anthropogenic disturbances are excluded.

In addition, we compared our estimates of biomass C storage with previous studies for similar temperate forest types. Zhu et al. [14] and Zhou et al. [15] reported that in northeastern China biomass C storage of coniferous and broadleaved mixed forest ranged from 182.7 and 191 Mg·C·ha^−1^; Smithwick et al. [30] found that biomass C storage reached 506–627 Mg·C·ha^−1^ in the temperate forests of the American Pacific Northwest; Pregitzer & Euskirchen [37] reported that average global temperate forest biomass C was 114.7 Mg·C·ha^−1^; while this study estimated biomass C storage of temperate forest ranged from 58.9 to 386.5 Mg·C·ha^−1^ across stand ages in NE China. These discrepancies in biomass C storage among those studies are probably related to stand age structure and human disturbance [4], [31], [37].

In our study, patterns of C storage in tree, understory, forest floor and soils differed within boreal forests but not with temperate forests (Table 4). Tree C contribution to the total C storage in the temperate mature and over-mature mixed forests ranged from 43.8% to 72.8% (Fig. 3), respectively, which was higher than that observed in the boreal mature and over mature forests (41.1% and 62.8%, respectively). Similar tree C proportions have been reported by others [14], [30]. The higher tree C proportion in temperate forests may reflect the effects of the relatively high growth temperatures in the temperate zone, leading to higher net primary production than that in the boreal zone [28]. As mentioned above, forest floor C is determined by the balance between litter input and decomposition output, where the latter is positively correlated with low temperature [34]. Thus, forest floor C accounted for a relatively greater proportion of total forest C storage (6.5%–12.0%) in the boreal forests (Fig. 3). This emphasizes the importance of the forest floor in maintaining the C pool in that forest zone [1], [37].

### Effect of Stand Age on Carbon Storage

Our study provides comprehensive estimates of forest C storage for tree, understory, forest floor and soil components and their distributional patterns on a regional scale. We found that stand age significantly influences tree C and total ecosystem C (Tables 3 and 4). As stand age increases, tree biomass C storage also increases (Fig. 2a–e). This result highlights the longer temporal accumulation of net primary production and the important role of tree biomass C in determining a forest ecosystem’s potential for carbon storage [32]. Thus the high tree biomass of over-mature forests (Fig. 2a–e) also portends a high potential for young, mid-aged and mature forests to sequester C.

Compared to increases tree C storage with stand age, C storage for the other observed forest components remained relatively stable with increasing stand age. The exception was forest floor C in boreal forests (Fig. 2k, l; Table 4). There, soil C, apparently remains in place over years to centuries [17], [44]. Similarly, previous studies also have failed to find significant differences in soil C storage among forests of different ages [18], [45]. In upland ecosystems, soil C storage is primarily determined by the balance between carbon input from litter production and output through decomposition [46]. But that balance can be affected by logging and other human disturbances, which in turn depend on the amount of biomass removed, quality of debris remaining, and soil fertility [16]. Martin et al. [17] reported that harvesting may not necessarily affect total soil C content, which suggests that soil C tends to remain relatively stable with increasing stand age provided that forests are not severely disturbed [15], [47].

The various forest components (tree, understory, forest floor and soil) have different C turnover times, and thus play different roles in C sequestration [1], [37]. For example, tree C contributions to total C storage increases with increasing stand age (Fig. 3). A greater proportion of tree C with respect to total C storage in mature and over-mature forests emphasizes the importance of maintaining mature and over mature forests [30]. In contrast, the soil C pool accounted for a greater proportion of total C storage in young and mid-aged forests than in old growth forests, reflecting a decreasing contribution of soil C to the total C storage with increasing stand age (Fig. 3) [48]. Thus there is an apparent shift in forest C partitioning as stands age. Over-mature forests maintained substantial C pools in both forest zones. Similarly, previous studies have shown that undisturbed old-growth forests continue to function as C sinks [47], [49], even though their rates of C incorporation into soil layers are low [45]. Hence, these observations have implications with respect to protecting mature and over-mature forests from human disturbance, limiting CO_2_ emissions, and ultimately global warming.

### Conclusion

Our study represents an early step in understanding the carbon pool size and its distribution varies among different ecosystem components, and across stand ages in the forests of NE China. We found that total forest C storage was greater in the temperate than in the boreal forests, as well as in older than in younger forests. Tree biomass C was the main component of total forest C storage, and its fraction increased with increasing stand age. This supports the great C sequestration potential of old-growth forests as observed by Zhou et al. (2006) and Luyssaert et al. (2008). Tree biomass C was significantly affected by forest zone (temperate vs. boreal), stand age, and forest type, which in turn are associated with climate, biomass accumulation rate, and stand composition and diversity. The large fraction of soil C within the total forest C storage indicated that soil C storage is also an important C pool in the forests of NE China. On the other hand, understory and forest floor C storages jointly contributed to <13% in boreal and <5% in temperate forests play a minor role to total forest C storage in NE China.
